# Thermography in Anesthetic Peripheral Nerve Blocks When Using Different Local Anesthetics

**DOI:** 10.3390/diagnostics15212743

**Published:** 2025-10-29

**Authors:** Andrejs Ernests Zirnis, Aleksejs Miščuks, Iveta Golubovska, Valērija Kopanceva, Everita Binde, Valentīna Sļepiha, Uldis Rubīns

**Affiliations:** 1 Faculty of Medicine and Life Sciences, University of Latvia, LV-1004 Riga, Latvia; aleksejs.miscuks@lu.lv (A.M.); kkopanceva@gmail.com (V.K.); 2 Hospital of Traumatology and Orthopedics, LV-1005 Riga, Latvia; 3 Faculty of Medicine, Riga Stradiņš University, LV-1007 Riga, Latvia; 4 Institute of Atomphysics and Spectroscopy, University of Latvia, LV-1004 Riga, Latvia; uldis.rubins@lu.lv

**Keywords:** regional anesthesia, peripheral nerve block, thermography, skin temperature, local anesthetics

## Abstract

**Background:** Peripheral nerve blocks in regional anesthesia are operator-dependent and are not always successful, leading to patient discomfort and postoperative pain. Current methods for assessing block failure rely on subjective patient reports of sensory and motor loss, which take time to appear and can be misleading. This study evaluates thermography as an objective, quantitative method for determining nerve block success and discusses its practical implications for clinical practice. **Methods**: This study was conducted at the Hospital of Traumatology and Orthopedics in Riga and included 55 patients undergoing sciatic nerve block with equipotent doses of different local anesthetics. Three local anesthetics—lidocaine, bupivacaine, and ropivacaine—were used in equipotent doses. After the block, the anesthetized region was imaged with a thermographic camera for 45 min to detect temperature changes. **Results**: Analysis showed no clinically significant differences among the local anesthetics in the timing or magnitude of skin temperature changes. At least 15 min must elapse before using thermography to judge nerve block success. Thermography is less reliable in acute bone fractures. Additionally, lower initial skin temperature was associated with a faster observable temperature increase, proving a strong negative correlation.

## 1. Introduction

Despite ongoing advancements in regional anesthesia—with new nerve block techniques and approaches regularly described in the literature and conferences—the methods we, as practicing anesthesiologists, use to determine nerve block success have remained largely unchanged for the past century. Currently, success is primarily assessed through subjective observations, including patient-reported sensations, the onset of motor blockade, and the loss of sensation in the targeted regions. These signs can take time to manifest, and patients may struggle to accurately evaluate or communicate their feelings, risking undetected failed blocks and resulting in pain during or after surgery.

The Ischiadic nerve block, performed via proximal or distal approaches, has long proven to be a valuable and effective technique for analgesia in surgeries involving the distal lower leg region. It significantly reduces pain and opioid consumption—especially when combined with a perineural catheter [1]—and can be paired with femoral or saphenous nerve blocks for complete anesthesia of the distal lower limb [2]. Compared to ankle blocks, it provides greater pain relief—averaging a 1.5-point more reduction on pain scales—and longer duration (up to 20 h versus approximately 14.5 h) [3]. Experienced operators using ultrasound guidance achieve success rates of 90–95% with the supra-popliteal approach [4], but failure still occurs in about 5–10% of cases, aligning with Barrington et al.’s retrospective study indicating approximately 10% failure among general peripheral nerve blocks [5].

Multiple factors contribute to nerve block failure. Well-documented predictors include avoidance of ultrasound guidance [6], less experienced operators [7], and higher body mass index (BMI), which complicates anatomical visualization and needle placement [8]. Other factors such as anxiety, nerve block location, anatomical variations, choice of local anesthetic, tourniquet pain, uncooperative patients [9], and male gender [8] have also been described. The success of a nerve block can also indirectly be improved with warming ropivacaine and bupivacaine anesthetics to 30 °C or more [10,11,12] and using injection pressure monitoring, which also serves as a precaution towards intraneural injections [13].

When multiple risk factors are present, it challenges even experienced practitioners. Strategies like ultrasound guidance, warming anesthetics, patient sedation, understanding tourniquet pain mechanisms, optimizing ultrasound imaging, and senior operator assistance can mitigate some risks. However, anatomical variations, male gender, uncooperative patients, and increased BMI-related challenges will always persist. In such complex cases, early and accurate detection of nerve block failure is vital, enabling timely intervention through rescue blocks, alternative regional approaches, or conversion to general anesthesia.

Thermography presents a promising solution for early detection of peripheral nerve block (PNB) failure. This non-invasive, quantitative technique analyzes skin temperature changes objectively—consistent, same picture evaluation across various independent observers—unlike the subjective and often unreliable clinical assessments [14]. Its potential application is currently limited by practical challenges, including the need for further validation and integration into routine practice.

Although thermography is not new and has been studied in regional anesthesia [15], broader clinical application remains limited. The purpose of this study, conducted at the Traumatology and Orthopedics Hospital, was to assess how different local anesthetics influence skin temperature changes following a popliteal sciatic nerve block, monitored via thermographic imaging. The primary objective was to determine how various anesthetics affect the speed of thermographic skin temperature changes. Secondary goals included analyzing the data for clinical relevance and evaluating thermography’s feasibility as a tool for detecting PNB failure in patients with acute bone fractures.

## 2. Materials and Methods

### 2.1. Study Design

From 30 November 2024 to 30 April 2025, a prospective randomized study was conducted at the Traumatology and Orthopedics Hospital in Riga. The study was approved by the Hospital’s Ethics Committee (application No. 49/2024/1) and followed the Declaration of Helsinki.

Both acute and elective surgical patients were enrolled. All participants were scheduled for surgery on the distal lower extremity and, according to international guidelines and the Hospital of Traumatology and Orthopedics’ best-practice recommendations, had indications for an ischiadic nerve block for postoperative analgesia. Additional inclusion and exclusion criteria are listed in Appendix A.1.

Patients were randomized into three groups according to the local anesthetic used (Groups B, R, and L). Each group comprised 20 patients. Randomization was performed using a free online randomization tool. Group B received 20 mL of 0.25% bupivacaine; Group R received 20 mL of 0.375% ropivacaine; Group L received 20 mL of 1% lidocaine.

Doses and concentrations were selected based on literature regarding the relative potency of each local anesthetic to ensure equipotency [16]. All eligible patients received full written and verbal information and provided informed consent. In addition, each patient signed the hospital’s standard consent form for anesthesia and surgery. Preoperative oral premedication included etoricoxib 90 mg and midazolam 7.5 mg or 3.75 mg (dose adjusted for age and comorbidities) for anxiolysis. Patients were assigned study numbers from 1 to 60 and allocated to one of the three randomized groups.

### 2.2. Procedure

Upon arrival, the surgical site was exposed to the environment up to knee level for 15 min to ensure thermal equilibrium, according to international medical thermography guidelines [17]. The ambient environment temperature in the preoperative room was maintained at 22 °C throughout the study. After 15 min, a baseline thermographic image was captured with a HICKMICRO SP60 camera (Hangzhou Hikmicro Sensing Technology Co., Ltd., Hangzhou, China). A suprapopliteal ischiadic nerve block was then performed using both ultrasound guidance and a nerve stimulator. Each patient received the assigned fixed dose of 20 mL of local anesthetic.

**Figure 1:**
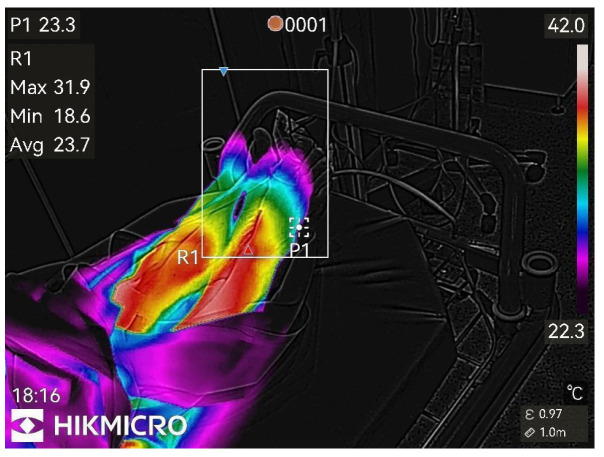
Before peripheral nerve block.

**Figure 2:**
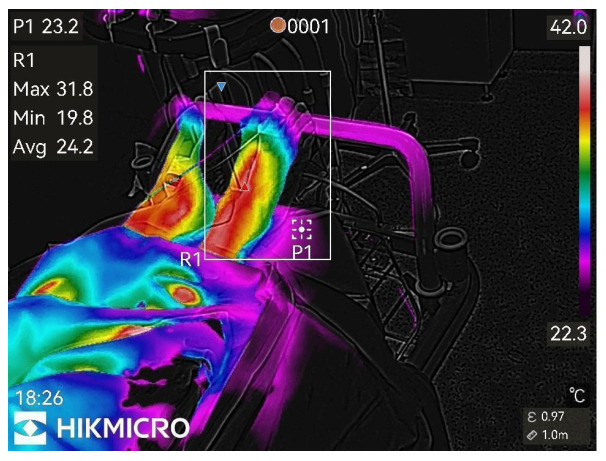
1st min after nerve block.

**Figure 3:**
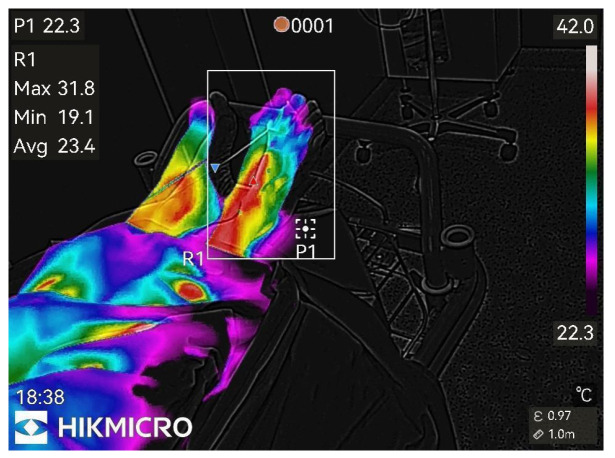
13th min after nerve block.

**Figure 4:**
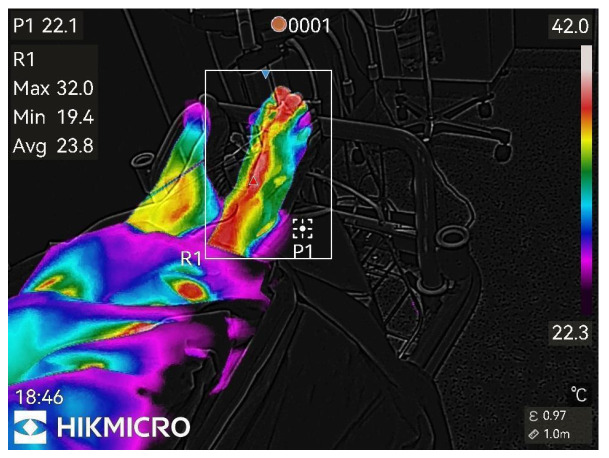
20th min after nerve block.

**Figure 5:**
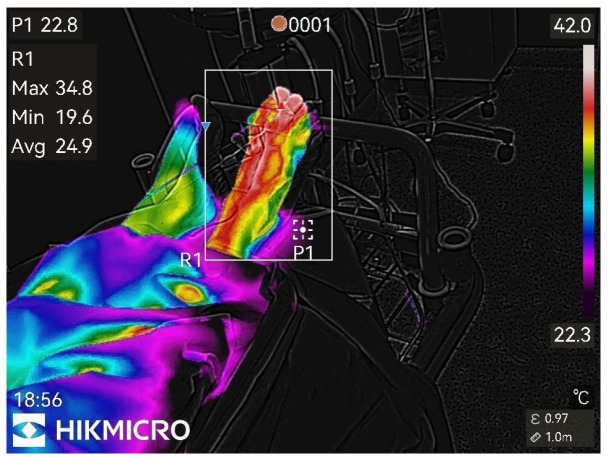
30th min after nerve block.

**Figure 6:**
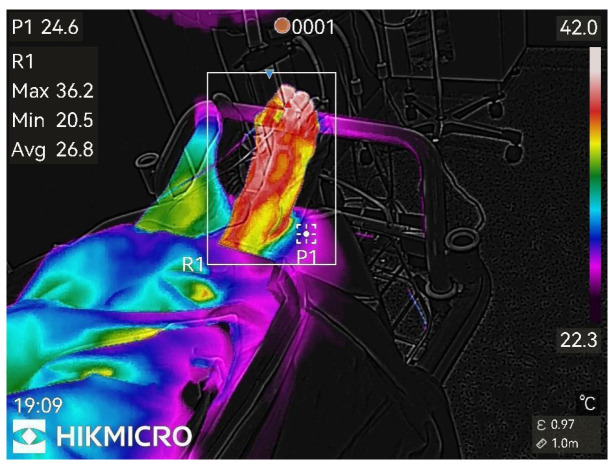
45th min after nerve block.

Thermographic images of the anesthetized limb were recorded immediately after the block and then every minute for 45 min (47 images per patient). Representative thermographic images from a single elective case (bupivacaine) are shown in Figure 1, Figure 2, Figure 3, Figure 4, Figure 5 and Figure 6.

A skin thermometer was attached to the toe of the anesthetized limb to provide continuous temperature measurements. Sensory testing with ice on the dorsum of the foot was performed every 3 min until cold sensation was absent. These measures served as additional confirmation of block success; the primary criterion for success was visible local anesthetic spread around the nerve within the Vloka fascial sheath on ultrasound.

### 2.3. Statistical Analysis and Data Storage

Thermographic images were saved on the camera and uploaded to Google Drive, then analyzed with the HICKMICRO Analyzer v1.7.1 thermography software. Data were recorded in Microsoft Excel (Microsoft 365). Statistical analyses were performed in IBM SPSS Statistics 29.0 (International Business Machines Corporation (IBM), Armonk, NY, USA) by the Statistics Laboratory at the University of Latvia, Riga.

Wilcoxon range tests, Spearman rank correlations were used to compare quantitative variable data. Linear regression methods were used for scalar reactions and variable correlations. A *p*-value < 0.05 was considered statistically significant.

Figure pronounced changes.

Almost maximal temperature changes, end of study.

## 3. Results

### 3.1. Total Population

During the study period, 55 of the planned 60 patients were enrolled; 7 were subsequently excluded due to inconclusive nerve blocks or poor-quality thermographic images. In the end, a total of 48 patients were analyzed.

**Figure 7:**
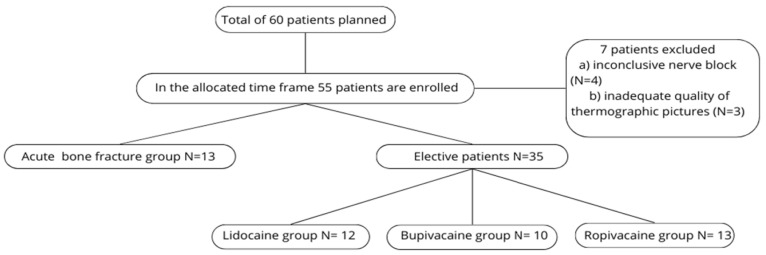
Flowchart of patient population.

For analysis, these 48 patients were divided into two main groups: patients with acute bone fractures (N = 13) and elective surgery patients without acute fractures (N = 35). The elective cohort was further divided into three subgroups based on the anesthetic used (Group L, Group B, Group R). Because the acute-fracture cohort was small, its three anesthetic groups were combined and analyzed as a single group (see Figure 7—flowchart).

### 3.2. Primary Outcome

To assess differences in the rate of skin temperature rise between the three local anesthetics (LAs), we used linear regression. Linear regression models the relationship between a scalar response and one or more explanatory variables; here, it quantified the correlation between time and temperature increase. A larger slope indicates a steeper curve and therefore a faster temperature rise per unit time.

The coefficients of determination (R^2^) for the fitted regressions were high across all groups, particularly for data up to minute 25—when temperature increases were most rapid—indicating that the models predict temperature change with high accuracy (see Appendix A.2). Over the full 45 min observation period, average skin temperature increases were relatively similar across groups. The slope coefficients for the full 45 min period were: ropivacaine SC = 0.194, lidocaine SC = 0.178, and bupivacaine SC = 0.162.

Examining only the first 25 min revealed larger differences: ropivacaine showed a substantially higher slope (SC = 0.315) compared with bupivacaine (SC = 0.234) and lidocaine (SC = 0.246). These findings are illustrated in the graphs in Appendix A.3 and Appendix A.4.

### 3.3. Secondary Outcomes

A key clinical question is how long it takes for thermography to detect statistically significant skin temperature changes after a peripheral nerve block. To answer this question, we used Wilcoxon signed-rank tests. Taking a significance level of α = 0.05 or less, the shortest time when the first statistically significant changes appear in the lidocaine group is 15 min, in the bupivacaine group—15 min, and in the ropivacaine group—10 min. It is worth noting that in the lidocaine group, the α value was already very close to 0.05 (0.057) at 10 min (Appendix A.5). This is consistent with the previously observed results for linear regressions, where the average temperature increase in the ropivacaine group was the most rapid, followed by the lidocaine and bupivacaine groups. If we use these obtained time points for statistically significant temperature changes, we can estimate the degree of temperature increase that will be observed at these cut-off times.

Estimated temperature increase at those cut-offs (relative to pre-block):

- Ropivacaine at 10 min: 3.70 °C
- Lidocaine at 10 min: 3.39 °C
- Bupivacaine at 10 min: 2.28 °C

(See Appendix A.6.)

We also examined whether baseline skin temperature affects the speed of temperature increase. Spearman’s rank correlation showed a strong, significant negative correlation between pre-block temperature and average rate of temperature change (ρ = −0.855, *p* < 0.001), meaning that the lower the initial skin temperature, the faster the observed temperature increase (Appendix A.8).

Lastly, Wilcoxon signed-rank range tests were used for the acute bone fracture group. Due to the small total number of patients in this group (N = 13), separate subgroups were not identified. An attempt was made to answer only one main question: Will a statistically significant change in temperature be observed in cases of acute bone fractures? All patients in this group had an initial skin temperature higher than 32 °C in the region of interest. Taking a significance level of α = 0.05 or less, the shortest time when the first statistically significant changes appear is 25 min. This confirms that statistically significant temperature changes will also be observed in acute bone fractures after peripheral nerve block (PNB) using the thermography method (Appendix A.7).

## 4. Discussion

Thermography presents an attractive alternative to commonly used diagnostic tests. It has already been studied in various peripheral and central nerve blocks.

For our study, after reviewing the literature and considering the specifics of our hospital workflow, we selected the ischiadic nerve block via the supra-popliteal approach as the preferred method, as thermography has already demonstrated efficacy in this type of block [18].

It is well known that each local anesthetic has its own onset time and duration. Literature indicates that lidocaine has the fastest onset, followed by ropivacaine and bupivacaine. These differences are based on the pharmacodynamic properties of these drugs—pKa, lipophilicity, and molecular size. However, both onset time and duration are also influenced by other factors, primarily the concentration and total volume of local anesthetic administered around the nerve [19]. To accurately assess how different local anesthetics affect the rate of skin temperature change, this study standardized these two factors by using a fixed volume and equipotent concentrations of local anesthetics.

Our analysis included only patients in whom the nerve block was considered successful, determined by the following parameters: convincing local anesthetic distribution around the nerve as confirmed by ultrasound, positive sensory blockade evidenced by ice testing (loss of temperature sensation), and an increase in temperature around the toe, continuously measured with a non-invasive skin thermometer. Only when all 3 criteria were met was a block deemed successful.

Based on the available literature, it was anticipated that lidocaine would produce the fastest onset of action and, consequently, the quickest temperature increase, followed by ropivacaine and bupivacaine [20].

Using linear regression analysis, our study found that the fastest temperature increase within 45 min occurred in the ropivacaine group (slope coefficient—SK = 0.194), followed by the lidocaine group (SK = 0.178) and the bupivacaine group (SK = 0.162).

When considering only the first 25 min, a more pronounced difference emerged: the slope coefficient for ropivacaine was significantly higher than for the other two anesthetics (SK = 0.315), compared to bupivacaine (SK = 0.234) and lidocaine (SK = 0.246). In this case, it could be explained that the true equipotent concentration of ropivacaine would be 0.33%, not 0.375%, which we used in the study. Nonetheless, the close similarity of the slope coefficients for lidocaine and bupivacaine indicates that the rate of temperature change is likely more influenced by concentration and volume (“strength”) rather than pharmacodynamic factors like pKa, etc.

Spearman’s rank correlation revealed a strong, statistically significant negative correlation between pre-block skin temperature and the mean rate of temperature change (ρ = −0.855, *p* < 0.001). This negative correlation aligns with previous findings that effective temperature increases after nerve blocks require an initial vasoconstriction in the anatomical region [2].

A clinically relevant question is: how long must pass after a nerve block before a statistically significant change in skin temperature can be observed?

It would be more meaningful to also view these changes in comparison with the previous different author study findings of the onset of sensory changes following the nerve block. Kim HJ et al. in their study using 0.375% ropivacaine and 0.25% levobupivacaine in infraclavicular blocks found that sensory block with ropivacaine occurs on average in 15–22.5 min, while levobupivacaine takes 17.5–35 min [21]. Cuvillon et al., who used various combinations of local anesthetics—including ropivacaine and bupivacaine at higher doses—found sensory-motor block onset times of approximately 23 ± 12 min for ropivacaine and 28 ± 12 min for bupivacaine [22]. Notably, their dosages were much higher (150 mg of ropivacaine and 100 mg of bupivacaine) than those used in our study.

Lidocaine used to be a popular choice in combinations with other local anesthetics for PNB, as it was believed it could enhance the speed of motor and sensory blockade. In modern regional anesthesia practice, it has fallen out of favor for this purpose after it was proven that it deteriorates the quality of nerve blocks while only modestly improving the speed of onset for sensory-motor changes [23] and due to concerns regarding neurotoxicity [24]. Regardless, some research using lidocaine admixture properties is still performed, such as the study done by Gamal M et al. They used a 1:1 mixture of 0.5% bupivacaine and 2% lidocaine in supraclavicular nerve blocks and measured changes with a thermographic camera in the relevant areas of the hand. It was found that a significant increase in temperature was observed in virtually all nerves after 15 min, meaning that in almost all cases, a successful nerve block could be assessed after 15 min [25].

Similarly, Asghar S. et al. studied thermographic changes after infraclavicular axillary nerve blocks, noting that a temperature difference of less than 30 °C between the 2nd and 5th fingers at 30 min indicated a failed block in all cases [26].

In our study, we used the Wilcoxon rank tests to determine at which point in time we could observe statistically significant temperature changes. Choosing a significance level of α = 0.05 or less, the shortest time when the first statistically significant temperature changes appear is 15 min in the lidocaine group, 15 min in the bupivacaine group, and 10 min in the ropivacaine group. It is worth noting that in the lidocaine group, the α value was already very close to 0.05 (0.057) at 10 min. In terms of absolute temperature increase figures, the total temperature increase for ropivacaine at 10 min will be 3.709 °C compared to the pre-block temperature, for lidocaine at 10 min it will be 3.392 °C, while bupivacaine will have the smallest increase, only 2.28 °C at 10 min (3.72 °C at 15 min). In short, our results correlate with those obtained by Gamal M et al.

In the acute fracture group, using Wilcoxon rank-sum tests and assuming a significance level of α = 0.05 or less, the shortest time at which the first statistically significant changes appeared was 25 min. This finding aligns with previous studies—specifically, the higher the initial temperature, the slower the temperature increase. In all cases of acute fractures, the initial temperature was 32 °C or higher. Der Strasse et al., who used a thermographic camera to diagnose acute bone fractures in a hospital emergency department, observed that in acute, combined lower leg fractures, the temperature difference between the two legs can be as high as 4.5 °C. This is consistent with the high skin temperatures observed in our study [27]. Since some initial temperatures were nearly 34 °C or higher, the overall temperature increase was minimal and inconclusive, partly because the maximum skin temperature cannot exceed a human core temperature of 36.6 °C.

To the author’s knowledge, this is the first study to investigate how different local anesthetics affect skin temperature changes and onset times in peripheral nerve blocks (PNB), using the same nerve block techniques, equipotent local anesthetic concentrations, and assessing the feasibility of methods for detecting PNB failure in cases of acute bone fractures. If this method is ever adopted for routine clinical use, addressing these key questions will be crucial.

## 5. Conclusions

The study found no clinically significant differences in skin temperature increases between thermography and three equipotent doses of local anesthesia (LA). The rate of temperature rise with these doses is primarily influenced by the initial skin temperature in the anesthetized area. Consequently, using this method to diagnose failed nerve blocks in acute bone fractures is not recommended, as the high initial vasodilation at fracture sites results in elevated baseline temperatures, making subsequent temperature increases minimal or undetectable. Lastly, at least 15 min should elapse after a nerve block before assessing its failure based on local anesthetic concentrations used in this study.

## Data Availability

The data presented in this study are available upon request from the corresponding author.

## Abbreviations

The following abbreviations are used in this manuscript:

SC	slope coefficient
PNB	peripheral nerve block
LA	local anesthetic
RA	regional anesthesia
P/o	per os

## Appendix A

### Appendix A.1. Inclusion and Exclusion Criteria

**Table A1:** Inclusion and Exclusion Criteria.

Inclusion Criteria	Exclusion Criteria
Surgery for the distal part of the lower leg	Patient’s refusal
Age 18–65	Repeated surgery on the lower extremity
Body Mass Index 23–38	Nerve damage (trauma or other)
	Decompensated diabetes (HbA1C > 8.5%)
	Peripheral artery disease (Fontaine 2 or above)
	Local infection in the region of surgery
	Patients with sepsis
	Bodyweight under 50 kg
	Chronic kidney insufficiency 3B or higher degree

### Appendix A.2. Table Shows Linear Regression Models for All 3 of the Local Anesthetics Groups

**Table A2:** Linear regressions.

Time	Free Member	Slope Coefficent	R2
Lidocaine, till 45th minute	28.46	0.178	0.94
Bupivacaine, till 45th minute	28.74	0.162	0.926
Ropivacaine, till 45th minute	28.53	0.194	0.879
Lidocaine, till 25th minute	27.73	0.246	0.986
Bupivacaine, till 25th minute	27.94	0.234	0.986
Ropivacaine, till 25th minute	27.24	0.315	0.967

This table shows R-squared values and slope coefficients for all elective patient cohorts. Additionally, on the lower part, results only till the 25th min were analyzed and showed much steeper slope coefficients with higher R-squared values.

### Appendix A.3. Linear Regression Models for Different Local Anesthetics up till 45 min

Colours: B—bupivacaine, L—lidocaine, R—Ropivacaine. Broken line refers to average temperature in groups, straight line of the same colour is linear regression of the same group.

### Appendix A.4. Linear Regression Models for Different Local Anesthetics up to the 25th min

Colours: B—bupivacaine, L—lidocaine, R—Ropivacaine. Broken line refers to average temperature in groups, straight line of the same colour is linear regression of the same group.

### Appendix A.5. Wilcoxon Range for Test Elective Patient Cohort

**Table A3:** Wilcoxon range tests.

Local Anesthetics/Minutes	0	5	10	15	20	25	30	35	40	45
Lidocaine	0.419	0.198	0.057	0.013	0.003	0.001	0.000	0.000	0.000	0.000
Buivacaine	0.650	0.570	0.290	0.026	0.003	0.002	0.001	0.001	0.001	0.001
Ropivacaine	0.939	0.356	0.010	0.002	0.001	0.000	0.000	0.000	0.000	0.000

The upper line represents time in minutes after nerve block, where 0 is the first minute right after, and 45–46th minute after nerve block. The rest of the numbers represent α values.

Taking a significance level of α = 0.05 or less, the shortest time when the first statistically significant changes appear in the lidocaine group is 15 min, in the bupivacaine group—15 min, and in the ropivacaine group—10 min.

### Appendix A.6. Temperature Changes in Absolute Numbers over Time

**Table A4:** Absolute temperature increase over time.

Local Anesthetics/Minutes	0	5	10	15	20	25	30	35	40	45
Lidocaine	0.825	1.267	1.300	1.833	0.475	0.858	0.800	0.550	0.275	0.217
Buivacaine	0.380	1.040	0.860	1.440	1.460	0.730	0.480	0.310	0.240	0.160
Ropivacaine	−0.177	1.369	2.523	1.577	0.931	1.000	0.538	0.354	0.038	0.200

The upper line represents time in minutes after nerve block, where 0 is the first minute right after and 45–46th minute after nerve block. The rest of numbers represent absolute temperature increase in degrees.

This table shows the absolute increase in temperature every 5 min, starting from 0 as the first minute after nerve block, i.e., in the Lidocaine group at 0 (first minute), skin temperature increased by 0.825 °C, at 5 (6 min)—for an additional 1.267 °C, in total summary increase during this time is 2.092 °C.

### Appendix A.7. Wilcoxon Range Tests for Acute Bone Fracture Group

**Table A5:** Wilcoxon range tests for Acute Bone Fracture Group.

Group/Minutes	0	5	10	15	20	25	30	35	40	45
Acute bone fractures	1	0.426	0.1	0.09	0.058	0.024	0.016	0.012	0.09	0.008

The upper line represents time in minutes after nerve block, where 0 is the first minute right after and 45–46th minute after nerve block. Statistically significant changes (α = 0.05 or less) can be seen after 25th minute.

### Appendix A.8. Rate of Skin Temperature Changes, Based on the Initial Temperature

This graph shows how initial skin temperature influences the speed of subsequent temperature increase after nerve block. Each black dot represents a single case from our study.
